# Effect of boron deficiency on the photosynthetic performance of sugar beet cultivars with contrasting boron efficiencies

**DOI:** 10.3389/fpls.2022.1101171

**Published:** 2023-01-16

**Authors:** Xin Song, Baiquan Song, Jialu Huo, Huajun Liu, Muhammad Faheem Adil, Qiue Jia, Wenyu Wu, Abudukadier Kuerban, Yan Wang, Wengong Huang

**Affiliations:** ^1^ National Sugar Crops Improvement Center & Sugar Beet Engineering Research Center Heilongjiang Province & College of Advanced Agriculture and Ecological Environment, Heilongjiang University, Harbin, China; ^2^ Research Institute of Economic Crops, Xinjiang Academy of Agricultural Sciences, Urumqi, Xinjiang, China; ^3^ Zhejiang Key Laboratory of Crop Germplasm Resources, Department of Agronomy, College of Agriculture and Biotechnology, Zhejiang University, Hangzhou, China; ^4^ Safety and Quality Institution of Agricultural Products, Heilongjiang Academy of Agricultural Sciences, Harbin, China

**Keywords:** Beta vulgaris L., boron deprivation, chlorophyll fluorescence, gas exchange indices, ultrastructure

## Abstract

Boron (B) deficiency severely affects the quality of sugar beet production, and the employment of nutrient-efficient varieties for cultivation is a crucial way to solve environmental and resource-based problems. However, the aspect of leaf photosynthetic performance among B-efficient sugar beet cultivars remains uncertain. The B deficient and B-sufficient treatments were conducted in the experiment using KWS1197 (B-efficient) and KWS0143 (B-inefficient) sugar beet cultivars as study materials. The objective of the present study was to determine the impacts of B deficiency on leaf phenotype, photosynthetic capacity, chloroplast structure, and photochemical efficiency of the contrasting B-efficiency sugar beet cultivars. The results indicated that the growth of sugar beet leaves were dramatically restricted, the net photosynthetic rate was significantly decreased, and the energy flux, quantum yield, and flux ratio of PSII reaction centers were adversely affected under B deficiency. Compared to the KWS0143 cultivar, the average leaf area ratio of the KWS1197 cultivar experienced less impact, and its leaf mass ratio (LMR) increased by 26.82% under B deficiency, whereas for the KWS0143 cultivar, the increase was only 2.50%. Meanwhile, the light energy capture and utilization capacity of PSII reaction centers and the proportion of absorbed light energy used for electron transfer were higher by 3.42% under B deficiency; KWS1197 cultivar managed to alleviate the photo-oxidative damage, which results from excessive absorbed energy (*ABS/RC*), by increasing the dissipated energy (*DI_o_/RC*). Therefore, in response to B deprivation, the KWS1197 cultivar demonstrated greater adaptability in terms of morphological indices and photosynthetic functions, which not only explains the improved performance but also renders the measured parameters as the key features for varietal selection, providing a theoretical basis for the utilization of efficient sugar beet cultivars in future.

## Introduction

Boron (B) is a metalloid and a beneficial element for maintaining the structural integrity of cell walls, which can reduce the uptake and accumulation of harmful substances in crops, thereby alleviating physiological damage caused by abiotic stresses (Qin et al., 2021; Qin et al., 2022; Yan et al., 2022). Negative alterations in these aforementioned physiological processes lead to a significant reduction in leaf area as well as biomass, retarded plant growth, and declined yield quality (Wang et al., 2015; Wei et al., 2022). Boron deficiency in soils is a widespread problem in agricultural production. Currently, B deficiency has led to reductions in yields of 132 crops in over 80 countries (Latifi and Jalali, 2018; García-Sánchez et al., 2020). Moreover, the exogenous application of B was reported to enhance dry matter synthesis and accumulation, which leads to an increase in crop yield (Zhou et al., 2013; Ahmed et al., 2020). Additionally, B plays a vital role in photosynthesis and transportation of the photoassimilates (Sharma and Ramchandra, 1990; Mishra et al., 2018). Boron deficiency is known to cause the impairment of leaf structure and function of the plant, resulting in reduced photosynthetic pigment content, reduced CO_2_ assimilation rate, and modulated blade expansion (Mukhopadhyay et al., 2013; Cong et al., 2015).

The cultivated land in China has a deficiency of B spreading across 33 million hectares. Sugar beet is an economic crop in northern China with a high B requirement. Low B-containing soils inevitably affect yield and sugar content, leading to lower economic returns from sugar beet cultivation and seriously limiting the development of the sugar industry in China. Different crop genotypes vary in their B requirements and utilization capacity (Pommerrenig et al., 2018). Employing B-efficient crops can effectively ameliorate the environmental risks associated with B administration and may help to promote sustainable agricultural development. Earlier studies on screening B-efficient grape cultivars (*Vitis vinifera* L.) have been reported as early as 1941 (Scott, 1941). Currently, the identification of B-efficient genotypes, with in-depth knowledge of physiological and molecular mechanisms, has been widely carried out in plants such as cotton (*Gossypium* spp) (Wu et al., 2018), oilseed rape (*Brassica napus* L.) (Yang et al., 2013), citrus (*Citrus sinensis* Obs) (Liu et al., 2017) and other horticultural crops.

In previous studies, 57 sugar beet cultivars grown worldwide for B utilization efficiency were identified (Song et al., 2021a; Wu et al., 2021; Song et al., 2022), and have obtained sugar beet B-efficient cultivars. Nevertheless, it is still unclear whether the physiological traits of B-efficient sugar beet cultivars correlate with leaf photosynthetic parameters under B-deficient conditions. The study investigated the changes and associations of leaf growth parameters, chloroplast ultrastructure, photosynthetic rate, and chlorophyll fluorescence (ChlF) parameters in sugar beet cultivars differing in B-efficiency under B deficit. Moreover, the study demonstrated how B-efficient cultivars responded to B stress, leading to changes in the leaf morphology, structure, and photochemical efficiency in different cultivars. This study will provide a theoretical basis for the utilization of B-efficient sugar beet cultivars in future breeding programmes.

## Materials and methods

### Plant material and growth indices

The current study was carried out at the experimental field of Heilongjiang University, Harbin (longitude: 126.61°, latitude: 45.71°), Heilongjiang, China. The B-efficient (H) genotyped sugar beet cultivar KWS1197 and B-inefficient (L) genotyped cultivar KWS0143 were selected from a previous study conducted by (Song et al., 2021a). The plants were cultivated in an artificial climate chamber (24°C/20°C). The sugar beets were cultured in 2 L containers and the roots were kept in the dark. The relative humidity was 65% and the photoperiod of seedlings was maintained 12:12 L to D; detailed information on the nutrient solution is presented in **Table 1** (Hoagland and Arnon, 1950). The plants were cultivated in the quarter-strength nutrient solution (25 μM H_3_BO_3_) in the first 7 days, then transplanted with half-strength nutrient solution at 7-day intervals. The plants were cultured in B-sufficient (50 μM H_3_BO_3_, B50) and B-deficient (0.1 μM H_3_BO_3_, B0.1) nutrient solution from the 7^th^ day (Song et al., 2021b); each treatment had three replications. The sugar beets were harvested at 28 d after treatment. The fresh weight (FW) was measured and oven dried to a constant weight at 60°C and dry weight (DW) to obtain the following parameters.

**Table 1:** The nutrient solution composition used in this study.

Nutrient solution composition	Concentration (L)
Ca(NO_3_)_2_.4H_2_O	5 mM
KNO_3_	5 mM
MgSO_4_.7H_2_O	2 mM
K_2_HPO_4_	0.18 mM
KH_2_PO_4_	0.20 mM
Fe-EDTA	10 μM
CuSO_4_.5H_2_O	0.31 μM
(NH_4_)_6_MO_7_O_24_	0.44 μM
MnCl_2_	4.45 μM
ZnSO_4._7H_2_O	0.76 μM


Leaf mass ratio (LMR)=leaf weight/total plant weight

Leaf mass fraction (LMF)=leaf weight/plant weight of shoot

Leaf area root mass ratio (LARMR)=total leaf area/total root mass

Leaf specific weight (SLW)=specific leaf area/dry weigh of specific leaf weight

Relative growth rate (RGR), net assimilation rate (NAR) and mean leaf area ratio (LARm) were determined according to the methods of Poorter (1999).

### Measurement of gas exchange parameters

The net photosynthetic rate (*P_n_*), stomatal conductance (*Gs*), transpiration rate (*T_r_*), and intercellular CO_2_ concentration (*C_i_*) of fully expanded leaves were measured by the LI-6400 (LI-COR., Lincoln, NE, USA) photosynthesis measurement system. *P_n_*/*T_r_* was used as the water use efficiency (WUE). portable photosynthesis measurement system. All measurements were performed between 9am and 12am, with relative humidity of 50–70%, CO_2_ concentration of 400 μmol mol^−1^, air temperature of 25°C to 28°C and photosynthetic photon flux density of 144 μmol m^−2^ s^−1^.

### Measurement of chlorophyll pigments

The chlorophyll pigment contents were determined according to the method described by Li et al. (2022). The fresh leaves (0.2 g) were cut and immersed in 10 mL of 95% ethanol (48 h) and the absorbance values were measured at 665 nm, 649 nm, and 470 nm.


$$\text{Chl }a\text{ concentrations }({\text{mg L}}^{-1})=13.95{\text{A}}_{665}-6.88{\text{A}}_{649}$$

$$\text{Chl }b\text{ concentrations}:\text{ Cb }({\text{mg L}}^{-1})=24.96{\text{A}}_{649}-7.32{\text{A}}_{665}$$

$$\text{Carotenoids concentrations}:{\text{ C}}_{\text{x}}\cdot_{\text{c}}({\text{mg L}}^{-1})=\frac{1000A470-2.05Ca-114.8Cb}{245}$$

The content of chlorophyll pigments in the leaf was calculated by the following formula:


$$\text{Pigment content }(\text{mg }{\text{g}}^{-1})=\frac{Pigment\;concentration\times Extract\;volume\times Dilution\;factor}{Sample\;fresh\;weight}$$

### Transmission electron microscopy

The cell structure of leaves was analyzed according to the protocol standardized by Song et al. (2019). The blade sections (1mm×2mm) after immersing in 2.5% (v/v) glutaraldehyde solution (2 h), and washing with 0.1 M phosphate buffer (pH 6.8) for 15 min (3 times) at 4°C, were fixed in 1% (w/v) OsO_4_ for two hours, followed by immersion in ethanol. Post-embedding in Spurr resin, the samples were cut with an ultra-microtome and examined photographed under a transmission electron microscope (H-7500, Japanese).

### Measurement of ChlF traits

The OJIP fluorescence induction curves of seedlings with different B efficiency under B deficient treatment were determined using Handy PEA (Hansatech Instruments Limited, Norfolk, UK). The OJIP curves were normalized to O-P and O-J: between F_o_ and F_J_: *V*_t_ =(F_t_−F_o_)/(F_p_−F_o_), and the differences of samples to the CK (KWS0143 B0.1treatment); between Fo and F_I_: *W*_k_=(F_t_−F_o_)/(F_k_ −F_o_) and the differences of the CK (KWS0143 B0.1treatment), and IP phase: (F_t_−F_o_)/(F_I_−F_o_)−1=(F_t_−F_I_)/(F_I_−F_o_). All ChlF parameters were measured following the methods described by Ye et al. (2019) and Wang et al. (2022). For this particular parameter, there were 9 replicates per treatment which were taken. Detailed information on the ChlF parameters evaluated in this study is presented in **Table 2**.

**Table 2:** The ChlF parameters used in this study.

Fluorescence parameters	Description
***F_0_ ≅ F_50μs_ or F_0_ ≅ F_20μs_***	Minimum fluorescence
***F_m_ = F_P_***	Maximum fluorescence
***V_J_ = (F_2ms_ − F_0_)/(F_m_ − F_0_)***	Relative variable fluorescence at 2 ms
***V_I_ = (F_30ms_ − F_0_)/(F_m_ − F_0_)***	Relative variable fluorescence at 30 ms
***W_t_ = (F_t_ − F_0_)/(F_J_ − F_0_)***	Relative variable fluorescence for the normalization between F_0_ and F_J_
***W_K_ = (F_300 μs_ − F_0_)/(F_J_ − F_0_)***	Ratio of variable fluorescence at K-step to the amplitude F_J_ − F_0_
***V_K_ = (F_300 μs_ − F_0_)/(F_m_ − F_0_)***	Relative variable fluorescence at 300 μs
***DF_ABS_***	The driving force based on absorbing light energy
***PI_ABS_***	Photosynthetic performance index based on absorbed light energy
***PI_total_***	Total PI, measuring the performance up to the PSI end electron acceptors
***ABS/RC***	Absorption flux per RC Light energy absorbed by per unit reaction center (RC)
***TR_o_/RC***	Trapped energy flux per RC at t = 0
***ET_o_/RC***	Electron transport flux per RC at t = 0
***DI_o_/RC***	Dissipated energy flux per RC at t = 0
***ABS/CS_m_***	Absorption flux per leaf cross section (t=t_Fm_)
***TR_o_/CS_m_***	Trapped energy flux per leaf cross section (t=t_Fm_)
***ET_o_/CS_m_***	Electron transport flux per leaf cross section (t=t_Fm_)
***DI_o_/CS_m_***	Dissipated energy flux per leaf cross section (t=t_Fm_)
***RC/CS_m_***	Number of active reaction sites per leaf cross section (t=t_Fm_)
***φ_po_***	Maximum quantum yield of primary photochemistry Maximum photochemical efficiency(t=0)
***φ_Eo_***	Quantum yield for electron transport(t=0)
***φ_Ro_***	Quantum yield of electron transport from ${\text{Q}}_{\text{A}}^{-}$ to the PS1 end electron acceptors
***δ_Ro_***	Efficiency with which an electron can move from the reduced intersystem electron acceptors to the PSI end electron acceptors
***ψ_Eo_***	Probability (t=0) that a trapped exciton moves an electron into the electron transport chain beyond QA^−^

### Statistics analysis

Significant differences in physiological parameters across the treatments were analyzed by one-way ANOVA using SPSS 25.0 (SPSS Inc, Chicago, IL) software. The illustration figure was drawn using Figdraw (https://www.figdraw.com/static/index.html). The graphs and PCA were drawn using Origin 2022 (Origin Lab Corporation, Wellesley Hills, Wellesley, MA, United States), whereas significant and extremely significant differences were expressed by “*” (*P<*0.05) and “**” (*P<*0.01).

## Results

### Effect of B deficiency stress on growth parameters of sugar beet seedlings with contrasting B efficiency

**Figure 1:**
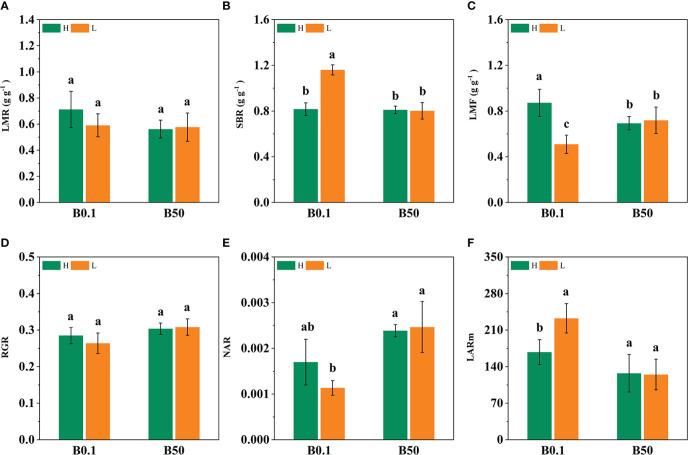
Effect of different B levels on growth paraments of two contrasting sugar beet cultivars (B-efficient: KWS1197**(H)** and B-inefficient: KWS0143 **(L)** cultivar). LMR is leaf mass ratio **(A)**. SBR is supporting organ biomass ratio **(B)**. LMF is leaf mass fraction **(C)**. RGR is relative growth ratio **(D)**. NAR is net assimilation ratio **(E)**. LARm is mean leaf area ratio **(F)**. Different letters (a, b, c) within a column represent significant differences at *P*<0.05. The seedlings were treated with B deficient (0.1 μM H_3_BO_3_, B0.1) and B sufficient (50 μM H_3_BO_3_, B50).

The development of different cultivars of sugar beet was inhibited by B-deficiency stress. The leaf mass ratio (LMR), support biomass of root (SBR), leaf mass fraction (LMF), and mean leaf area ratio (LARm), all showed an increasing trend under B deprivation. The LMR, LMF, and LARm of the KWS1197 sugar beet cultivar increased by 26.82%, 25.81%, and 56.15% respectively, while those of the KWS0143 cultivar increased by 2.50%, 11.32%, and 13.54% respectively. The increase was greater in the KWS1197 sugar beet cultivar than in the KWS0143 (**Figures 1A–C, F**). The RGR and NAR of the KWS1197 cultivar decreased by 9.17% and 41.91%, whereas for the KWS0143, it decreased by 13.60% and 24.13%, respectively (**Figures 1D, E**).

### Effect of B deprivation on photosynthetic and chlorophyll indices of different cultivars of sugar beet

**Figure 2:**
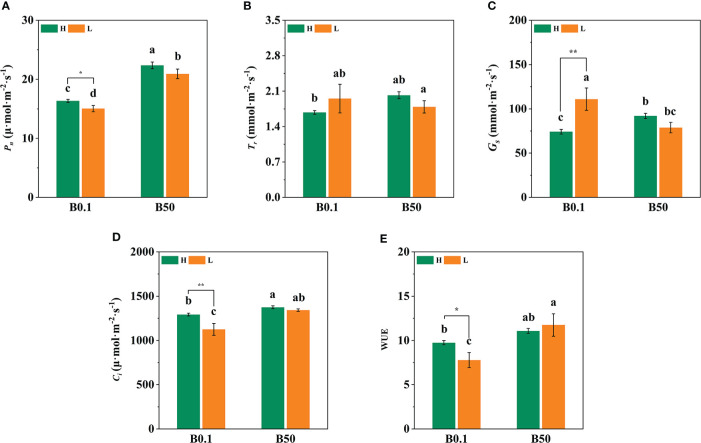
Effect of photosynthesis parameters in KWS1197 and KWS0143 cultivars. The seedlings were treated with B deficient (0.1 μM H_3_BO_3_, B0.1) and B sufficient (50 μM H_3_BO_3_, B50). P_n_ is the net photosynthetic rate **(A)**, *T_r_* is the transpiration rate **(B)**, *G_s_* is the stomatal conductance **(C)**, *C_i_* is the intercellular CO_2_ concentration **(D)** and WUE is the water use efficiency **(E)**. Different lowercase letters (a, b, c) indicated significant differences between different treatment. Bars denote the mean (n=3) and error bars the standard error.

**Figure 3:**
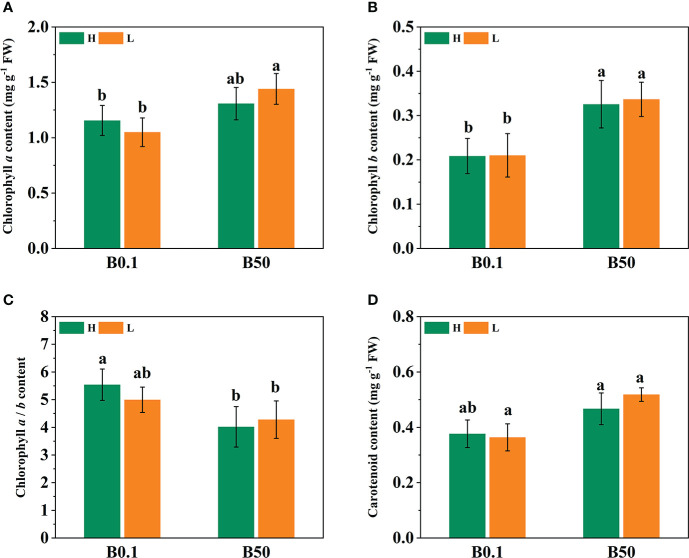
Effect of chlorophyll pigments in KWS1197 and KWS0143 cultivars. The seedlings were treated with B deficient (0.1 μM H_3_BO_3_, B0.1) and B sufficient (50 μM H_3_BO_3_, B50) treatments. The chlorophyll pigments include the content of chlorophyll *a* **(A)**, chlorophyll *b* **(B)** carotenoid **(D)** and the ratio of chlorophyll *a* to chlorophyll *b* **(C)**. Different lowercase letters (a, b, c) indicated significant differences between different treatments. Bars denote the mean (n=3) and standard error bars.

The *P_n_*, *T_r_*, *G_s,_* and *C_i_* of the two sugar beet cultivars’ foliage decreased (*P*<0.05) under B deficient growth conditions. Compared to the B50 treatment, *P_n_*, *T_r_*, *G_s_*, and *C_i_* of KWS1197 cultivar decreased by 15.29%, 22.84%, 26.84% and 12.08%, respectively (**Figure 2**). Moreover, the WUE of different sugar beet cultivars increased under B0.1 treatment; compared to the KWS0143 cultivar, the WUE was greater in the KWS1197 cultivar. The chlorophyll pigment content of sugar beet leaves showed no significant changes in response to B deprivation. Under the B0.1 treatment, the KWS0143 cultivars showed a reduction of 13.26%, 7.87%, and 5.75% in Chl *a*, Chl *b*, and Chl *a/b*, respectively. However, in the KWS1197 cultivar, the reduction in Chl *a*, Chl *b*, and Chl *a/b* was only 3.98%, 5.22% and 1.38%, respectively (**Figures 3A, B, D**). These results showed how the KWS1197 cultivars maintained the chlorophyll pigment system under low B stress conditions. Furthermore, in B-deficient conditions, the *car* content of sugar beet leaves increased by 2.03% in the KWS1197 cultivar, while a 10.61% decrease was recorded for KWS0143 cultivar (**Figure 3C**).

### Effect of B deprivation at the ultrastructural level

**Figure 4:**
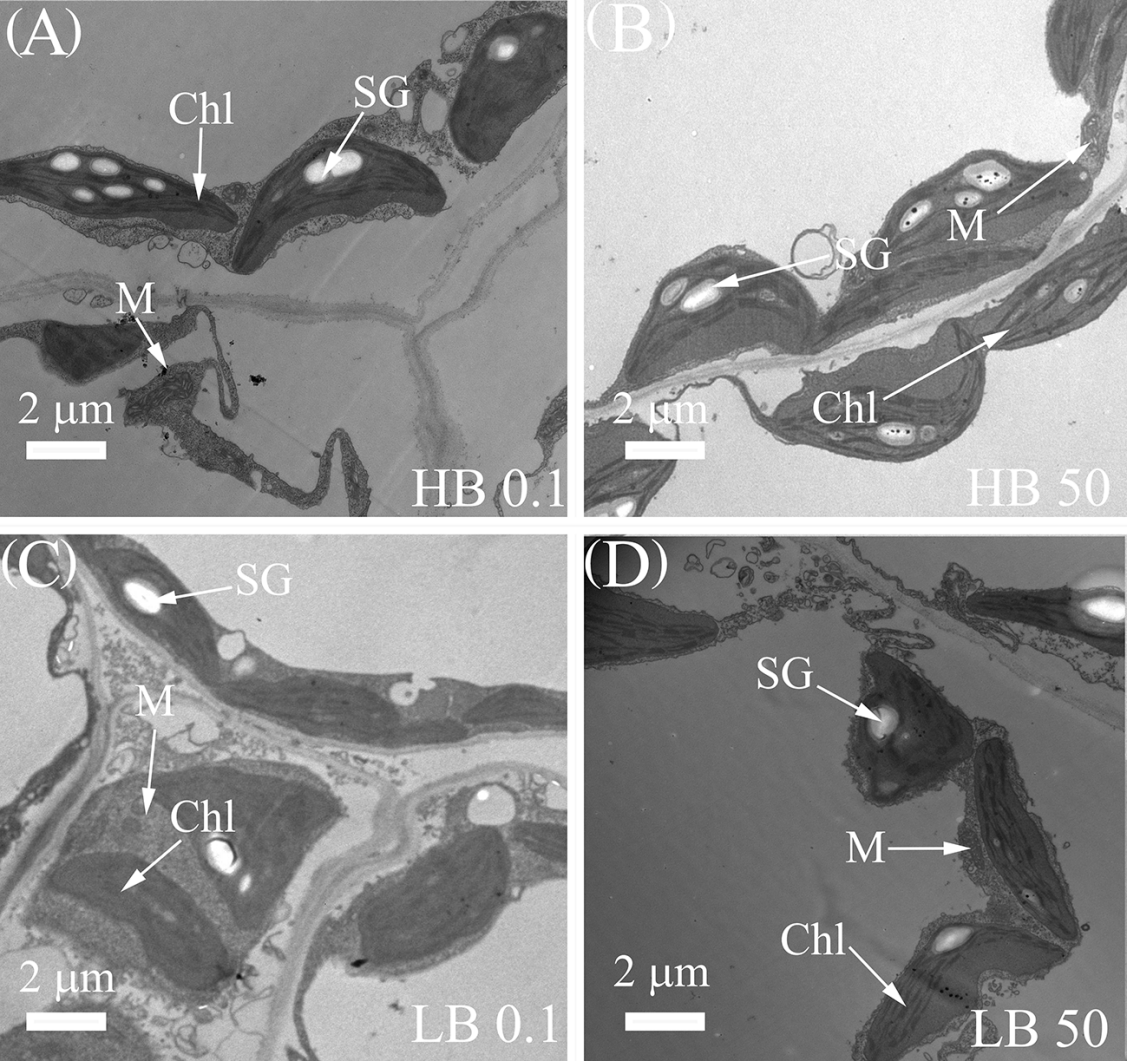
Changes in the subcellular structure of leaves of contrasting B efficiency sugar beet cultivars. The seedlings were treated with B deficient (0.1 μM H_3_BO_3_, B0.1) treatment in HB0.1 **(A)** and LB0.1 **(C)** and B sufficient (50 μM H_3_BO_3_, B50) treatments in HB50 **(B)** and LB50 **(D)**. Chl, Chloroplast; M, Mitochondrion; SG, Starch Granule.

The microscopic studies revealed that the internal structure of leaf chloroplast was retained under the B0.1 treatment in KWS1197. In terms of internal cellular structure, under B50 treatment, the cell structure of different cultivars of sugar beet leaves was intact, chloroplasts were well developed (**Figures 4B, D**), the lamellar structure was neatly arranged, and starch granules were visible. However, under B-deficient conditions, the intergranular thylakoid structure was loose, and the lamella was disordered (**Figures 4A, C**). Compared to the KWS0143 cultivar, the chloroplast lamellae of the KWS1197 sugar beet cultivar were more stable under B deficiency. There was no apparent disorder of the cystoid matrix compared to the KWS0143 cultivar sugar beet.

### Effect of B-deprivation on the leaf OJIP transients and energy allocation parameters of PSII of sugar beet with contrasting B efficiency

**Figure 5:**
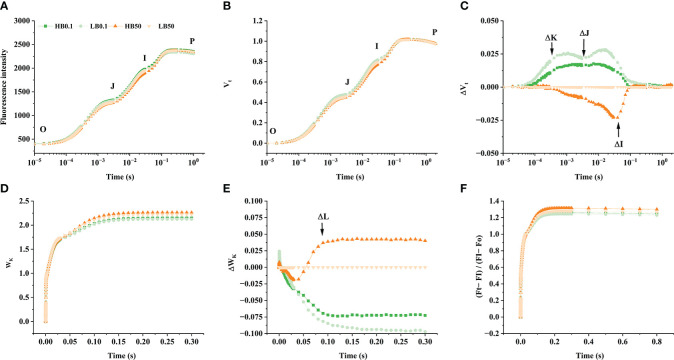
The effect of OJIP curve **(A)**, V_t_ **(B)**, ΔV_t:_ the difference of V_t_ **(C)**, W_k_ **(D)**, ΔW_k_: the difference of W_k_ **(E)** and (F_t_-F_I_)/(F_I_-F_0_) **(F)** of KWS1197**(H)** and KWS0143 **(L)** cultivars. The seedlings were treated with B deficient (0.1 μM H_3_BO_3_, B0.1) and B sufficient (50 μM H_3_BO_3_, B50) treatments.

**Figure 6:**
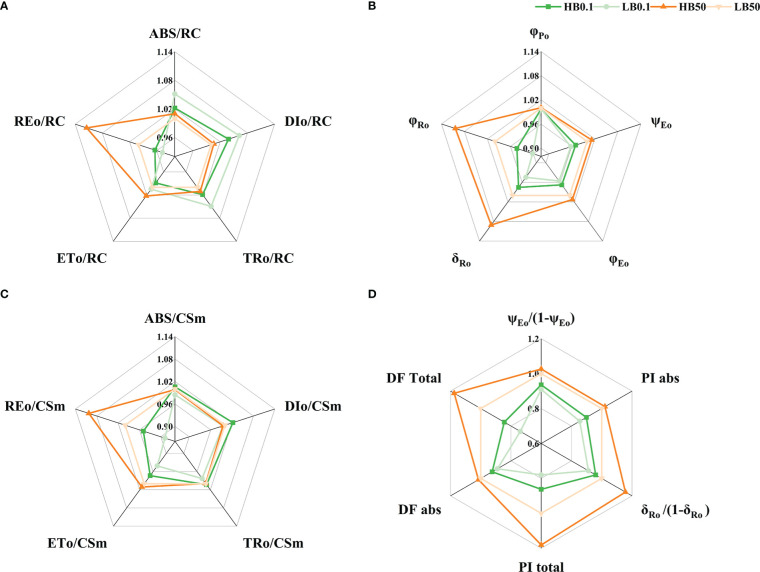
Relationships between parameters describing fluorescence and yield of photosystem II in KW1197**(H)** and KWS0143**(L)**cultivars. The seedlings were treated with B deficient (0.1 μM H_3_BO_3_, B0.1) and B sufficient (50 μM H_3_BO_3_, B50) treatments. The parameters of the energy flux of the PSII reaction center of reducible QA **(A)**, quantum yield and flux ratio **(B)**, phenomenological flux per unit leaf section (t= tFM) **(C)** and performance index **(D)**. Values are means (n=9) after standardization.

Boron-deficient conditions caused significant changes in the ΔV_t_ and ΔW_k_ curves of different KWS1197 sugar beet leaves (**Figure 5**). In the study of ΔV_t_ and ΔW_k_ curves, it was found that in ΔV_t_ curve, there were positive ΔK, ΔJ, and ΔI bands. The magnitude of change in the H sugar beet cultivar was less compared to the KWS0143 cultivar; there were ΔL-band in curve ΔW_k_. The OJIP curve, the V_t_ and W_k_ curves, and the (F_t_-F_I_)/(F_I_-F_0_) curve did not show significant changes under B deficit. During B deficiency stress, the *ABS/RC*, *TR_o_/Rc*, and *DI_o_/RC* were suggested to increase. The *ET_o_/RC* and *RE_o_/RC* decreased in the two sugar beet cultivars, reflecting increased energy uptake and dissipation in the PSII reaction center of individual reducible Q_A_. However, in B deficiency, the electron energy transferred and the electron flux transported to the terminal electron acceptor of PSI for reduction was decreased, moreover the KWS1197 cultivar was less affected than the KWS0143 cultivar (**Figure 6A**). The quantum yield to flux ratio of the leaves of both sugar beet cultivars was affected by B deficiency, and its φ_Po_, φ_Eo_, φ_Eo_/(1-φ_Eo_), φ_Ro_, ψ_Eo_, δ_Ro,_ and δ_Ro_/(1-δ_Ro_) were reduced, indicating that the maximum photochemical efficiency, quantum yield for electron transfer, and quantum yield of the terminal electron acceptor on the reduced PSI receptor side, were all reduced by B-deficient stress; the KWS1197 cultivar was reduced to a lesser extent than KWS0143 cultivar (**Figures 6B, D**). In B deficiency, the flux per leaf cross section of different sugar beet cultivars were adversely affected, in which *ABS/CS_m_* and *DI_o_*/*CS_m_* increased. At the same time, *RE_o_/CS_m_* and *ET_o_/CS_m_* decreased, while *TR_o_/CS_m_* indicated that the absorbed energy per unit leaf section increased under B deficiency. The energy flux captured by the PSII active reaction center was less affected when the transferred energy flux was reduced and heat dissipation increased (**Figure 6C**). In B-deficient conditions, *PI_ABS_*, *PI_total_*, *DF_ABS_*, and *DF_total_* were reduced in the leaves of both cultivars, and the overall functional activity of PSII, PSI, and inter-systemic electron transport chains, as well as the collective driving force, were reduced but to a lesser degree in KWS1197 cultivar (**Figure 6D**).

### Principal component analysis and correlation among the photosynthetic parameters

**Figure 7:**
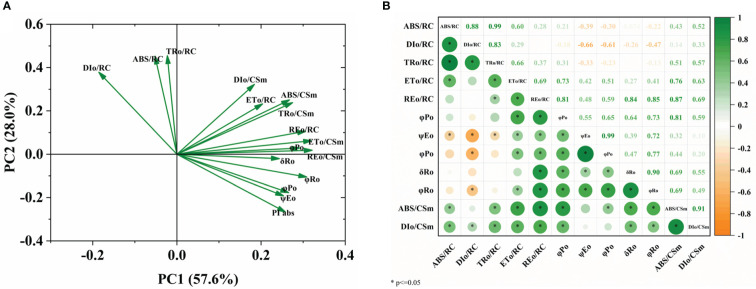
PCA of plant measured factors. **(A)** Loading plot, **(B)** correlation matrix. The plants were exposed to B deficient (0.1 μM H_3_BO_3_, B0.1) and B sufficient (50 μM H_3_BO_3_, B50) treatments.

The main factors of chlorophyll fluorescence (ChlF) parameters in different sugar beet cultivars were investigated using PCA. PC1 distinguished the energy flux of the PSII reaction center of reducible Q_A_ from the quantum yield or flux ratio and the image-only flux per unit leaf section. The highest contribution of image-only flux per unit lobe cross-section indicates that these factors largely measure energy absorption, transfer, and dissipation in the PSII reaction center of reduced Q_A_. PC2 distinguished the quantum yield from the energy flux in the PSII reaction center of reduced Q_A_ (**Figure 7A**). Correlation studies showed that the quantum yield was positively correlated with the *ET_0_/RC* and *RE_0_/RC* (**Figure 7B**).

## Discussion

**Figure 8:**
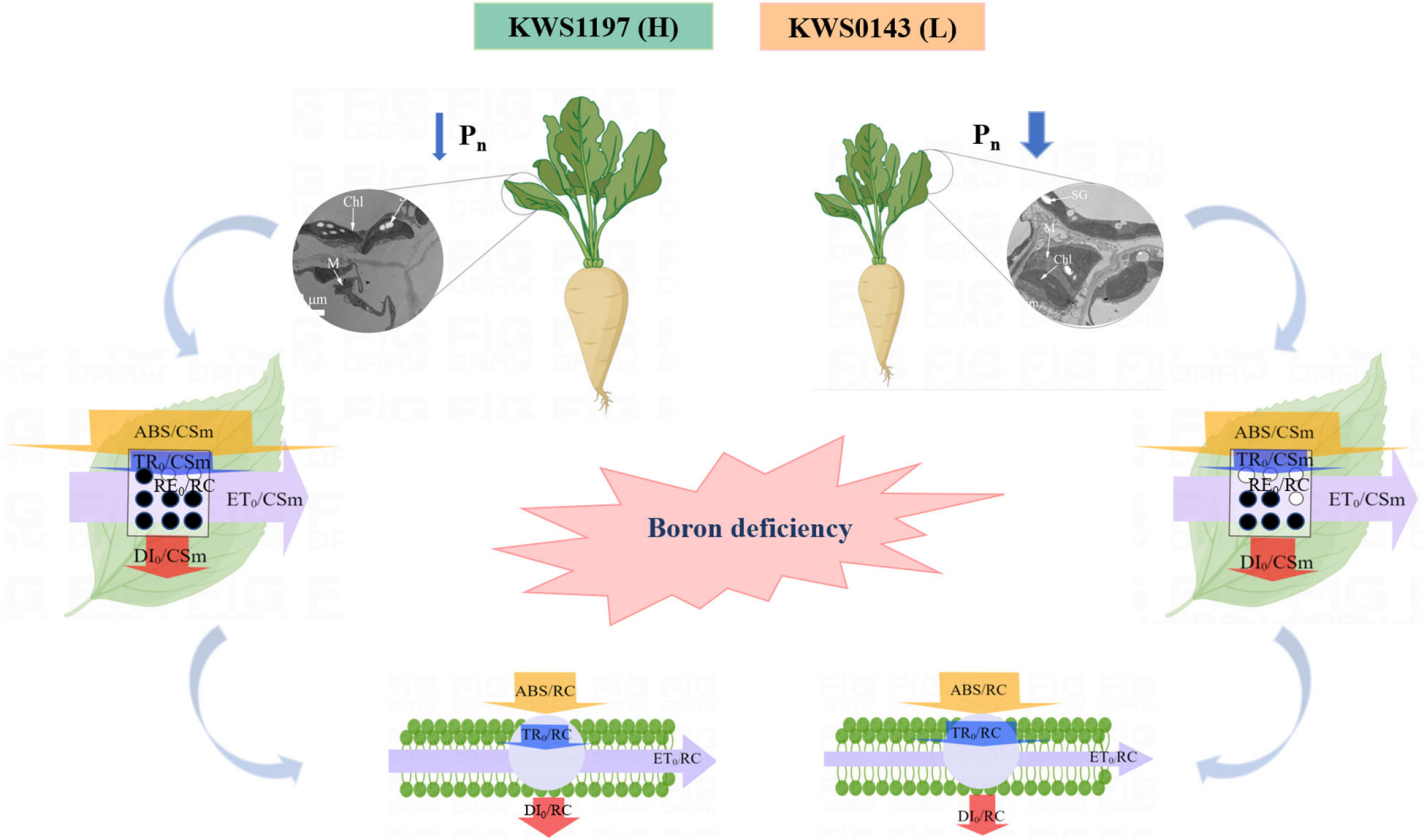
Photosynthetic physiological mechanism of efficient B utilization in seedling leaves of sugar beet cultivars.

In current study, the KWS1197 cultivar was more advantageous in terms of leaf phenotypic traits, photosynthetic performance, and energy utilization of the PSII (**Figure 8**). Boron deficiency caused increased LMR and insignificant SBR changes in the two tested sugar beet cultivars, ultimately leading to an improved ratio of assimilated to non-assimilated tissues and a reduced plant growth rate. Compared to the KWS0143 cultivar, the higher growth rate of the KWS1197 seedlings suggest that this sugar beet cultivar’s foliage had a greater adaptive response to the B-deficient environment. It has also been shown that B-deficient growth conditions significantly reduced biomass in *Sacha inchi* and *Neolamarckia cadamba* (Yamuangmorn et al., 2021; Yin et al., 2022). Furthermore, a study conducted on citrus plants demonstrated that B-efficient genotypes experienced less reduction in biomass under low B conditions (Liu et al., 2015). Contrastingly, in a study based on B-inefficient genotype of *Arabidopsis*, the growth rate was significantly reduced, and growth/development was significantly delayed under B deprivation (Pommerrenig et al., 2018), which remains consistent with the present findings. Furthermore, it was revealed that B-deficiency stress significantly impacted the *P_n_* of different sugar beet cultivar seedlings. However, the KWS1197 cultivar underwent lesser reduction and exhibited a rather greater net photosynthetic rate than the B-inefficient variety. Similarly, B deprivation was found to cause a decrease in *P_n_* in cabbage and cotton plants by means of Hill reaction’s inhibition and a decrease in *C_i_* (Choi et al., 2016). Previous studies have shown that B deficiency decreased gas exchange parameters in grapes, but the decrease was also smaller in B-efficient genotyped grape cultivars (Wei et al., 2022).

Some studies have found that B-deficient environments also affect photosynthetic pigments’ content. For example, B deficiency was found to reduce chlorophyll content in studies on citrus (Hua et al., 2017; Yan et al., 2022). Albeit, other studies have found an increase in chlorophyll content after short-term low B treatment (Chen et al., 2014). Conversely, a study on cotton found that chlorophyll content changed unremarkably when B-deficient stress reduced the *P_n_* of leaves (Liu et al., 2017). The current study found that under B-deficient conditions, the amount of photosynthetic pigment did not change significantly. It might be because the duration of B-deficient treatment and the degree of B-deficient stress were associated, indicating that the change in photosynthetic pigment content in this study was not the dominant factor causing the decline in leaf photosynthetic rate. On the other hand, the effect of B deprivation on the photosynthetic performance of plants was also reflected in the damage to photosynthetic organs. In citrus studies, it was revealed that chloroplast structure was also damaged in B-deficient stress, leading to reduced CO_2_ assimilation (Yan et al., 2022), and thus affecting photosynthetic rate (Yang et al., 2022). The present investigation established that grana were disorganized and structurally abnormal under B deficiency. Notably, compared to the KWS0143 cultivar, KWS1197 showed a complete chloroplast structure and neatly arranged lamellar structure of leaves.

It has been demonstrated earlier that B deficiency does not directly damage the PSII and impact photosynthesis, but rather the photoinhibition caused by B deficiency is associated with abnormal carbon metabolism (Han et al., 2008). The adverse effects of B deprivation on the photosynthetic system can be reflected by PSII, which eventually causes alterations in fluorescence. The current study showed that the positive ΔK-band indicated OEC deactivation, fewer antenna complex connections, and a distorted thylakoid membrane. Furthermore, it ultimately led to reduced energy transfer and uptake efficiency, which was also observed in studies of nitrogen-deficient as well as calcium-deficient crops (Aleksandrov et al., 2014; Cetner et al., 2017). Additionally, with B deficiency stress, the *ABS/RC* increased while *ET_o_/RC* and *ET_o_/CS_m_* decreased; the *DI_o_/RC* and *DI_o_/CS_m_* tended to increase, indicating that B deficiency increased the light energy absorbed while decreasing the energy used for electron transfer in sugar beet seedling leaves, which is consistent with the appearance of a positive ΔK-band. While the dissipated energy increased (*DI_o_/RC* and *DI_o_/CS_m_*), which in turn could reduce the excess excitation energy (*ABS/RC*) in PSII and positively protect the integrity of the electron transport chain. The KWS1197 cultivar’s photochemical efficiency was higher than the KWS0143. In citrus, light energy absorbed also increased because of B deficiency, which implies a partial inactivation of the reaction center (Han et al., 2009) and an increase in the energy dissipated to avoid photo-oxidative damage (Aleksandrov et al., 2014), a mechanism to protect damaged leaves. The same conclusion has been drawn in studies on *Lolium Perenne* L. (Zhang et al., 2018).

The leading site of impaired electron transport is the inactivation of the PSII receptor side, which indicates the occurrence of photo-inhibition damage (Ye et al., 2019). During B-deficient stress, ΔJ-band and ΔI-band appeared in different cultivars of sugar beet leaves, indicating that B deprivation affected the reduction of the PSII receptor side and oxidation of the PSI receptor side, resulting in the photo-inhibition. It became apparent that φ_Eo_, φ_Ro_ decreased in B deficiency (**Figure 6B**), demonstrating photochemical reactions on PSII for electron capture as well as transfer. The reduced efficiency of the photochemical reaction on PSII for electron capture and transfer, together with the reduced *PI_abs_*, reflects the impaired reduction of the terminal electron acceptors of PSI in sugar beet. The magnitude of change was less in the KWS1197 cultivar than the KWS0143. It was found in studies involving tomato seedlings that the light energy absorbed by the leaf PSII reaction center increased in the presence of reduced *RE_o_/CS_m_* (Kalaji et al., 2014). The same finding was established in the present study, which could be a protective mechanism for sugar beet seedlings to enable energy availability under B deficiency.

## Conclusion

The current study alluded that B deficiency retarded leaf growth and development of contrasting B efficiency sugar beet cultivars, reducing the photosynthetic rate and photochemical efficiency of the PSII. Meanwhile, inactivation of the PSII receptor side induced the onset of photoinhibition, limiting light energy transfer, and thus reducing the CO_2_ assimilation rate of sugar beet. The KWS1197 cultivar of sugar beet leaves in comparison with the KWS0143 cultivar under B deficient growth conditions had less reduction in *P_n_*. Meanwhile, the KWS1197 cultivar was suggested to have an accumulation of biomass, a better intact structure of thylakoid, higher energy transfer and absorption efficiency in PSII, a stronger activity of OEC and reaction center, higher light utilization efficiency, and was exposed to a less degree of photoinhibition. The advantage of the KWS1197 cultivar in photochemical utilization under B deficiency is one of the critical reasons for the difference under B efficient conditions, which enriches the theoretical basis for the effective use of B nutrition in sugar beet. The present study will help the breeders understand and select the B-efficient cultivars of sugar beet by screening photosynthetic parameters, which were shown to correlate with biomass and yield parameters.

## Data availability statement

The original contributions presented in the study are included in the article/supplementary material. Further inquiries can be directed to the corresponding authors.

## Author contributions

XS: Writing-review and editing. Investigation, Software. BS: Conceived and designed the experiments, Supervision and Funding acquisition. JH: Software and Writing review & editing. MFA and QJ and WW and WH: Writing-review & editing. HL and BS and AK and WH: Writing-review & editing and Funding acquisition All authors contributed to the article and approved the submitted version.
